# Medical and advanced heart failure therapies in Türkiye

**DOI:** 10.55730/1300-0144.5933

**Published:** 2024-05-07

**Authors:** Sanem NALBANTGİL, Emre DEMİR, Ahmet ÇELİK, İnci Tuğçe ÇÖLLÜOĞLU, Naim ATA, Mehmet Birhan YILMAZ, Anıl ŞAHİN, Dilek URAL, Mustafa Mahir ÜLGÜ, Emine Arzu KANIK, Lale Dinç ASARCIKLI, Yüksel ÇAVUŞOĞLU, Selda MURAT, Şuayip BİRİNCİ

**Affiliations:** 1 Department of Cardiology, Faculty of Medicine, Ege University, İzmir, Turkiye; 2 Department of Cardiology, Faculty of Medicine, Mersin University, Mersin, Turkiye; 3 Department of Cardiology, Faculty of Medicine, Karabük University, Karabük, Turkiye; 4 General Directorate of the Health Information Systems, Ministry of Health, Ankara, Turkiye; 5 Department of Cardiology, Faculty of Medicine, Dokuz Eylül University, İzmir, Turkiye; 6 Department of Cardiology, Faculty of Medicine, Sivas Cumhuriyet University, Sivas, Turkiye; 7 Department of Cardiology, Faculty of Medicine, Koç University, İstanbul, Turkiye; 8 Department of Biostatistics, Faculty of Medicine, Mersin University, Mersin, Turkiye; 9 Department of Cardiology, Faculty of Medicine, Health Sciences University, İstanbul, Turkiye; 10 Department of Cardiology, Faculty of Medicine, Eskişehir Osmangazi University, Eskişehir, Turkiye; 11 Deputy Health Minister, Ministry of Health, Ankara, Turkiye

**Keywords:** Device therapy, heart failure, medical therapy, nationwide study

## Abstract

**Background/aim:**

Effective management of heart failure involves evidence-based use of multiple medications and their combinations. Furthermore, dosage escalation of the recommended medications is advised. In cases of advanced heart failure, long-term mechanical assistance devices or heart transplantation surgery may be necessary. Socio-economic disparities create unequal opportunities for people to access these treatments.

The study aimed to analyze and compare medical and advanced heart failure treatments recommended by guidelines across various regions in Türkiye.

**Materials and methods:**

About 85 million citizens medical treatment records were utilized between January 1, 2016, and December 31, 2022. Medical and heart replacement treatment opportunities for heart failure in Türkiye were evaluated in the general population and across different geographical regions.

**Results:**

According to this study, beta-blockers were the most commonly prescribed medication for heart failure in Türkiye. This was followed by angiotensin-converting enzyme inhibitors at 44% and mineralocorticoid receptor antagonists at 38.9%. However, only 0.6% of patients used angiotensin receptor blocker-neprilysin inhibitors. Despite the high incidence of diabetes mellitus among heart failure patients, only 11% used sodium-glucose cotransporter two inhibitors. The study also found that using an implantable cardioverter defibrillator (ICD) was 0.8%, and cardiac resynchronization therapy (CRT) was 0.3% among all intracardiac device treatments. Heart replacement therapies, cardiac transplantation surgery, and long-term left ventricle-assisted device (LVAD) surgery had very low rates.

**Conclusion:**

The use of guideline-directed medical therapy is not optimal in Türkiye and varies across different geographical regions. It is a fact that heart transplant or LVAD surgery, CRT, and ICD implantation rates in Türkiye are significantly lower than those in developed countries, regardless of geographical region.

## 1. Introduction

In developed countries, the incidence of heart failure (HF) is decreasing due to better management of cardiovascular diseases. However, the overall incidence of HF is increasing due to an aging population [1–4]. Pharmacotherapy should be the first-line treatment for HF before considering nonpharmacological interventions and device therapy [5,6].

Current HF management guidelines advise the use of multiple medications for patients with HF, particularly in reduced ejection fraction, which has been proven to improve survival and quality of life [7]. To achieve maximal benefits, guidelines recommend that each evidence-based medication be titrated to the target dose as tolerated in the shortest time possible [7].

The modulation of the renin-angiotensin-aldosterone (RAS) and sympathetic nervous systems through the use of angiotensin-converting enzyme inhibitors (ACE-i) or an angiotensin receptor-neprilysin inhibitors (ARNI), beta-blockers, and mineralocorticoid receptor antagonists (MRA) has been proven to enhance survival, lessen the risk of HF hospitalizations, and reduce symptoms in patients with HF [8–10]. It is recommended to use a combination of an ARNI/ACE-i/ARB, a beta-blocker, and an MRA as the mainstay therapy. The sodium-glucose cotransporter 2 (SGLT2) inhibitors, when added to triple therapy including ACE-I/ARNI/beta-blocker/MRA, have been shown to minimize the risk of cardiovascular death and worsening HF in patients with HFrEF [11,12].

Despite the strong evidence derived from clinical trials, the prevalence of optimal medical therapy is often underestimated in patients with HF and the rate of achieving target doses in primary therapies is low. According to the VICTORIA registry, a study of patients hospitalized for chronic heart failure with reduced ejection fraction (HFrEF), a significant number of eligible patients were not prescribed certain medications at the time of discharge. Specifically, 33% were not prescribed a RAS inhibitor (RAS-i), 25% were not prescribed beta blockers, and 55% were not prescribed an MRA. Although approximately half of the patients had type 2 diabetes mellitus, less than 1% were prescribed an SGLT2 inhibitor on discharge [13].

A median of 0.26 (IQR: 0.11–0.57) hospitals per million people reported left ventricular assist device (LVAD) procedures with a median of 1.90 (IQR: 0.41–3.49) implants per million per year in Europe [14]. Moreover, heart transplant rate increased from 0.65 per million people in 2000 to 2.93 per million people in 2019 (annual procedure change: 9.9% [95% CI: 8.1–11.8]) in Europe [15].

This study aims to report the usage rates of evidence-based therapies and advanced HF treatments among patients with HF in Türkiye.

## 2. Materials and methods

Our study was a nationwide retrospective cohort study based on population data. The study protocol was approved by the Ministry of Health of Türkiye under approval number 95741342-020. The study design and procedures adhered to the Declaration of Helsinki guidelines. We used anonymized data from the National Electronic Database of the Turkish Ministry of Health. The database contained over 85 million citizen records between January 1, 2016, and December 31, 2022. Since 2016, the Turkish Ministry of Health has governed the national electronic database, merging all other databases, including procedures, laboratory results, medications, and deaths. It is mandatory for all hospitals to report data since 2016. Because all databases have been merged and are governed by the Turkish Ministry of Health, diagnoses can be cross-checked, for example, with compatible medications after index diagnoses or planned follow-up data for procedures. All drugs and medical equipment are digitally tracked and recorded into this database, except for those without reimbursement. We used the following International Statistical Classification of Diseases and Related Health Problems (ICD)-10 codes to identify patients with HF: I50.0 (congestive HF), I50.1 (left ventricular dysfunction), I50.9 (HF, unspecified), I11.0 (hypertensive heart disease with congestive HF), I13.0 (hypertensive heart and chronic kidney disease with congestive HF), I13.2 (hypertensive heart and chronic kidney disease with congestive HF and renal failure), and I42.0 (dilated cardiomyopathy).

The statistical analysis was done using SPSS 25.0 software (IBM Corp., Armonk, NY, USA) and E-PICOS AI (MedicReS, NY, USA). Continuous variables are expressed as mean ± standard deviation, median-interquartile range, and categorical variables as number and proportion.

## 3. Results

The research involved 2,722,151 patients with HF between 2016 and 2022. The aim of the study was to analyze and compare guideline-directed medical treatments for HF across different regions. The study revealed that beta-blockers were the most commonly prescribed medication for HF, followed by ACE inhibitors at 44% and mineralocorticoid receptor antagonists (MRA) at 38.9%.

It has been found that a mere 16% of those with HF use angiotensin receptor blocker (ARB) class drugs, and an even smaller number of 0.6% use angiotensin receptor blocker-neprilysin inhibitors (ARNI). In contrast, a significant 45.2% of patients with HF take oral antidiabetic medication, while 11% of patients use SGLT-2 inhibitors, regardless of their diabetic status.

**Figure 1:**
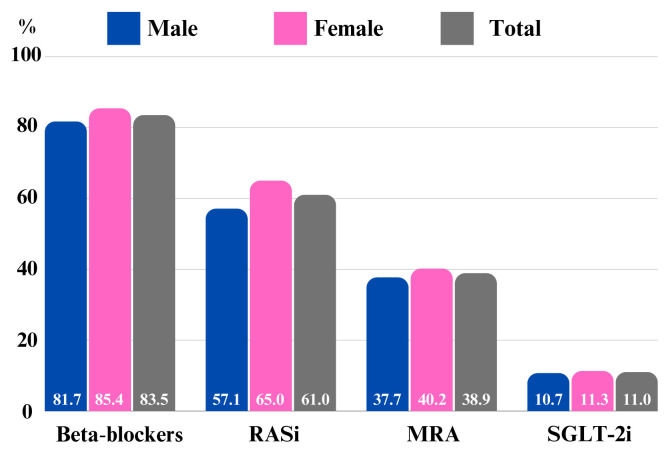
Usage rates of guideline-directed medical therapy for heart failure based on sex.

Based on the analysis of patient records, it was discovered that 17.4% of the subjects were prescribed warfarin, while 20.3% were given nonvitamin K oral anticoagulants (Table 1). For a breakdown of drug treatments administered to patients with HF based on sex, it has been observed that treatment based on evidence, such as beta-blockers, renin-angiotensin-aldosterone system blockers (ACE inhibitors, ARBs, ARNI), MRA, and SGLT-2 inhibitors, is more commonly used in men than in women among individuals with HF. This trend is visible and illustrated in Figure 1.

The use of drug treatments for HF has been evaluated separately in different geographical regions, and the results are presented in Table 2. The highest beta-blocker use is observed in the Southeastern Anatolia Region at 86.2%, while the lowest is in the Eastern Anatolia Region at 82.2%. The Marmara Region has the highest rate of ACE inhibitors at 47.6%, while the Black Sea Region has the lowest rate at 41.7%. The highest rate of ARBs is found in the Marmara Region at 17.4%, and the Eastern Anatolia Region has the lowest rate at 12%. The Aegean Region has the highest rate of MRAs at 41.5%, while the Eastern Anatolia Region has the lowest rate at 31.8%. The highest rate of SGLT-2 inhibitors is also found in the Aegean Region at 11.8%, and the lowest rate is in the Eastern Anatolia Region at 9.3%. Regarding digoxin usage, the Aegean Region has the highest rate at 16.8%, while the Eastern Anatolia Region has the lowest rate at 10.7%.

**Figure 2:**
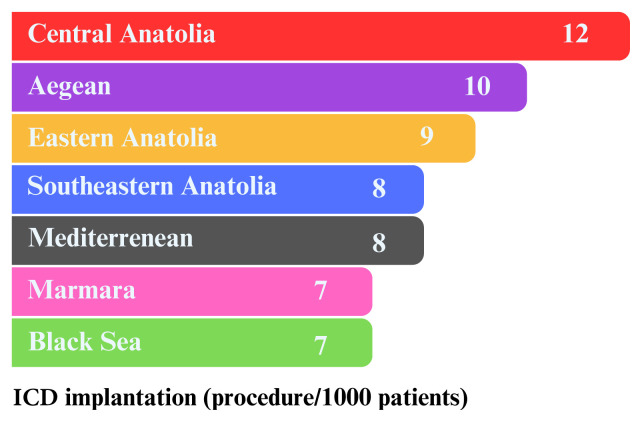
Geographical distribution of ICD implantation rates per 1000 patients.

**Figure 3:**
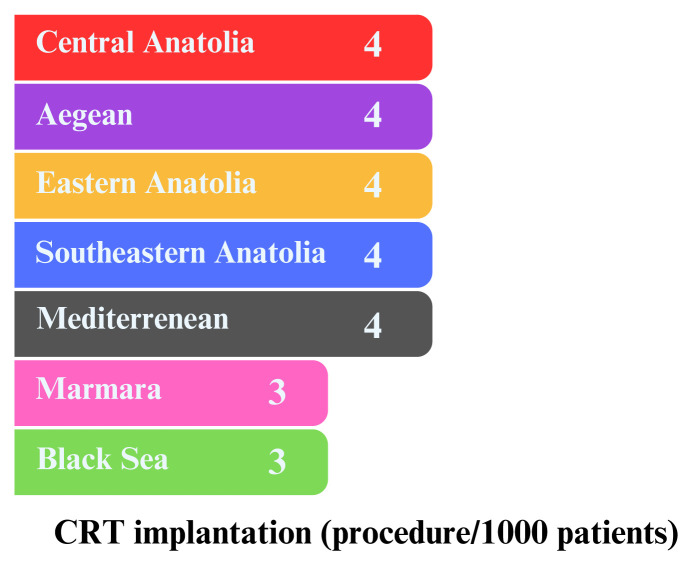
Geographical distribution of CRT implantation rates per 1000 patients.

Table 3 lists the advanced treatment methods available for HF patients in our country. The use of an implantable cardioverter defibrillator (ICD) was found to be 0.8%, and cardiac resynchronization therapy (CRT) was 0.3% among all intracardiac device treatments. Men were found to receive both these devices more frequently than women. Between 2016 and 2022, 148 patients underwent heart transplantation, including 38 women and 110 men. The Marmara Region has the highest number of transplants, followed by the Black Sea, southwestern, and Aegean regions. The east of Türkiye has the lowest rate of transplantation. The use of long-term mechanical support is highest in the southwest and Marmara regions. Ventricular assist devices were used more frequently in men (344) than in women (57) for treating advanced-stage HF patients. Transcatheter aortic valve implantation (TAVI), a treatment for severe aortic stenosis, was applied to 0.2% of all HF patients. Among these, 2368 female patients with HF were treated with TAVI, whereas only 1761 male patients received this treatment. Between 2016 and 2022, more men than women underwent cardiac and pulmonary rehabilitation programs. During this period, it was observed that more men (5.9%) received renal replacement therapy than women (4.5%). Patients with heart failure had low rates of coronary artery bypass surgery (2.5%), mitral valve replacement (0.3%), aortic valve replacement (0.3%), and percutaneous valve repair surgery (0.0%). The chart in Figure 2 illustrates the number of ICD implantations performed in Türkiye between 2016 and 2022. The data, categorized by geographical regions, shows that the Central Anatolia Region had the highest rate of ICD implantation, with 12 patients per thousand. In contrast, the Marmara and Black Sea regions had the lowest rate, with seven patients per thousand. Figure 3 displays the regional distribution of CRT implantations in Türkiye from 2016 to 2022. The Aegean and Marmara regions had the lowest CRT implantation rates, with three patients per thousand.

## 4. Discussion

This study provides a detailed evaluation of the drugs used to treat HF. However, the utilization rates obtained are for the entire patient group, regardless of ejection fraction (EF). The usage data of drug groups, beta-blockers, RAS blockers, MRA, and SGLT-2 inhibitors that have significant effects on survival, especially in the HFrEF patient group, appears to be relatively low.

Among these treatments, beta-blockers were found to be used most frequently in our country. According to world data, beta-blockers are the most frequently used drug group, especially HFrEF [16,17]. Although RAS blockers were used at relatively low rates, it should be considered that the study population included all HF groups independent of EF.

ARNI usage is significantly low in our country despite its mortality advantage when added to standard treatment in HFrEF patients [10,18]. The use of this drug in the right patient group produces valuable results. In our country, the number of patients who can access ARNI treatment is quite low because of its high cost and the fact that it is not yet covered by reimbursement. The results for using MRAs in our country are similar to world data, where MRAs are used by an average of 40% of HF patients [19].

SGLT-2 inhibitors are new drugs that have been used worldwide in treating HF. Studies have shown that they have positive effects on clinical outcomes in all HF groups, regardless of EF [20,21]. From 2016 onwards, they have been included in the treatment guidelines for patients with HFrEF, and from 2021, they will also be recommended for those with HFpEF. However, in our country, their usage rates are relatively low (11%), even though they are considered a promising new treatment for HF patients without diabetes mellitus. This is because SGLT-2 inhibitors are currently only reimbursed for patients with diabetes mellitus, which limits their use. If reimbursement conditions for HF patients without diabetes mellitus are changed, more patients may be able to access this treatment. Additionally, cardiologists are currently unable to prescribe these drugs, which may also contribute to their limited use. Addressing both reimbursement and prescription issues may increase the survival rates of HF patients in our country.

It is estimated that 37.1% of HF patients in our country have atrial fibrillation (AF), and around 33% receive oral anticoagulant treatment. This suggests that most patients are being treated with anticoagulants, which is important because the presence of AF alongside HF increases the risk of blood clots. However, drug use rates are lower in regions that are socioeconomically less developed. Therefore, it is crucial to ensure that HF treatment is applied correctly and adequately throughout the country, and more research is needed on this topic.

In addition to traditional medical treatments, advanced applications and device support may be necessary for treating HF. Devices such as ICDs and CRTs can prevent sudden death, but it is essential to select suitable patients for these treatments. Registry studies conducted in Europe have reported that the implantation rate of ICD is approximately 20%, and the CRT use rate is approximately 15% [9]. However, in our country, the rate of device treatments such as ICD and CRT is relatively low (0.8% and 0.3%, respectively) compared to data from all over the World. At the same time, there is a significant price difference between university hospitals and private hospitals. Additionally, interruptions caused by the supply of devices from abroad, lack of domestic production, and possible problems with physicians consulted by HF patients can also be some of the reasons for the low usage. The lack of awareness among people can also be a contributing factor.

In Türkiye, it has been reported that 17.7% of HF patients are accompanied by chronic kidney disease, and renal replacement therapies are applied to approximately 5.9% of patients with end-stage renal failure. Since people with chronic kidney disease are excluded from clinical drug trials, evidence-based information is scarce, particularly regarding the treatment of patients with GFR <30 mL/min. Drugs such as ACE-i, ARBs, ARNI, and MRA, which are known to reduce mortality in HF, are either not started or discontinued due to side effects such as worsening renal failure or hyperkalemia [22,23]. A similar lack of information also applies to renal replacement therapies, and the correct and rational use of renal replacement therapies in this fragile patient group accompanied by both heart and kidney failure is crucial. Therefore, research on the subject should be supported.

Around 5% of HF patients have advanced HF and require specialized treatments such as inotropic therapy, heart transplantation, or ventricular mechanical assist devices (VAD) [24]. Each year, heart transplantation is performed on over 4000 patients in the USA alone and over 2000 patients in Europe [25,26]. However, heart transplantation rates in our country are low primarily due to the insufficient number of donors. TRends-HF study from Türkiye found that the number of heart transplants performed in our country was significantly lower (148 patients) compared to worldwide data. Additionally, only 401 patients received left ventricular assist devices, primarily used as a bridge to transplant [27]. It is observed that the percentage of patients who receive a VAD and can access heart transplantation varies depending on various factors such as the stage of HF, general health status, suitability of the device, and cost. Studies conducted in the Western world have shown that approximately 38% of patients who received a VAD as a bridge to transplantation underwent heart transplantation [28]. In the United States, registry data spanning 30 years showed that the probability of heart transplantation within one year after VAD implantation was 47% [29]. However, in our country, the rate of subsequent heart transplantation in patients who undergo VAD implantation is relatively low, at 3%. Although heart transplantation and the use of ventricular assist devices require specialized teams and centers and are very costly procedures, the SSI reimbursement system in Türkiye covers these treatments. Intraaortic balloon pump is the only available short-term percutaneous mechanical support. Other temporary assist devices like TandemHeart and Impella are not being reimbursed in Türkiye. The number of patients with cardiogenic shock who benefit from this therapy is also very small when compared to European countries. ECMO is being implanted by specialized teams, and again, the number is very limited. Therefore, the main challenge is to provide suitable patients with these treatment methods and the right teams. This calls for establishing specialized centers for advanced-stage HF throughout the country. Additionally, comprehensive studies need to be carried out nationwide, and national incentive campaigns are necessary to eliminate donor shortage.

## 5. Conclusion

The use of guideline-directed medical and device therapy is not optimal in Türkiye and varies across different regions. The most commonly prescribed drug is beta blockers. The use of ARNI and SGLT-2 inhibitors is relatively low due to reimbursement issues. Implantation of ICD and CRT is less than in North America and Europe. Although the number of heart transplants is relatively low, durable assist device implantation is comparable in Europe. More tertiary heart transplant centers with specialized teams are mandatory for the management of advanced heart failure, especially in eastern Türkiye. To address the shortage of heart transplant donors and improve public awareness, it is imperative for the Ministry of Health to initiate targeted campaigns.

## Figures and Tables

**Table 1:** Heart failure medical therapies in Türkiye and comparison by sex.

	Male (n = 1,314,224)	Female (n = 1,407,927)	Total (n = 2,722,151)
**Beta-blockers, n (%)**	1,121,912 (85.4)	1,150,579 (81.7)	2,272,491 (83.5)
**ACE inhibitors, n (%)**	648,565 (49.3)	559,196 (39.7)	1,207,761 (44.4)
**ARB, n (%)**	194,858 (14.8)	239,803 (17.0)	434,661 (16.0)
**ARNI, n (%)**	11,616 (0.9)	5206 (0.4)	16,822 (0.6)
**MRA, n (%)**	529,300 (40.2)	532,481 (37.7)	1,058,504 (38.9)
**SGLT-2i, n (%)**	148,159 (11.3)	150,448 (10.7)	298,607 (11.0)
**Ivabradine, n (%)**	108,386 (8.2)	60,535 (4.3)	168,921 (6.2)
**Digoxin, n (%)**	171,116 (13.0)	238,056 (16.9)	409,172 (15.0)
**Statin, n (%)**	742,378 (56.5)	624,821 (44.4)	1,367,199 (50.2)
**Amiodarone, n (%)**	115,464 (8.8)	93,039 (6.6)	208,503 (7.7)
**Calcium channel blockers, n (%)**	592,298 (45.1)	796,364 (56.6)	1,388,662 (51.0)
**Acetylsalicylic acid, n (%)**	1,097,666 (83.5)	1,070,299 (76.0)	2,167,965 (79.6)
**Warfarin, n (%)**	213,526 (16.2)	260,749 (18.5)	474,275 (17.4)
**NOACs, n (%)**	230,390 (17.5)	322,236 (22.9)	552,626 (20.3)
**Oral antidiabetics, n (%)**	559,263 (42.6)	671,017 (47.7)	1,230,280 (45.2)

ARB: angiotensin receptor blockers; ARNI: angiotensin receptor blockers/neprilysin inhibitors; MRA: mineralocorticoid receptor antagonists; NOACs: nonvitamin K oral anticoagulants;RAS: renin-angiotensin system; SGLT-2i: Sodium-glucose cotransporter two inhibitors.

**Table 2:** Distribution of heart failure drug use rates in Türkiye by geographical region.

	Geographical regions						
	Mediterranean (n= 326,092)	Eastern Anatolia (n = 178,762)	Aegean (n = 382,725)	Southeastern Anatolia (n = 184,177)	Central Anatolia (n = 430,982)	Black Sea (n = 382,082)	Marmara (n = 828,963)
**Beta blockers, n (%)**	280,200 (85.9)	146,963 (82.2)	318,024 (83.1)	165,890 (86.2)	359,988 (83.5)	314,454 (82.3)	685,972 (82.8)
**ACE-i, n (%)**	140,779 (43.2)	75,354 (42.2)	161,913 (42.3)	88,126 (45.8)	187,788 (43.6)	159,463 (41.7)	394,338 (47.6)
**ARB, n (%)**	54,159 (16.6)	21,413 (12.0)	57,202 (14.9)	27,719 (14.4)	70,244 (16.3)	59,678 (15.6)	144,246 (17.4)
**ARNI, n (%)**	1876 (0.6)	495 (0.3)	2909 (0.8)	1300 (0.7)	1874 (0.4)	1864 (0.5)	6504 (0.8)
**MRA, n (%)**	132,791 (40.7)	56,890 (31.8)	158,958 (41.5)	78,074 (38.9)	175,532 (40.7)	141,190 (37.0)	318,696 (38.4)
**SGLT-2i, n (%)**	38,045 (11.7)	16,649 (9.3)	45,016 (11.8)	21,820 (11.3)	45,181 (10.5)	40,665 (10.6)	91,231 (11.0)
**Ivabradine, n (%)**	23,244 (7.1)	8173 (4.6)	29,707 (7.8)	11,713 (6.1)	21,835 (5.1)	25,770 (6.7)	48,479 (5.8)
**Digoxin, n (%)**	54,144 (16.0)	19,168 (10.7)	64,113 (16.8)	25,875 (13.4)	66,734 (15.5)	61,957 (16.2)	117,181 (14.1)
**Statins, n (%)**	163,635 (50.2)	85,010 (47.6)	177,169 (46.3)	96,170 (49.9)	227,964 (52.9)	187,701 (49.1)	429,550 (51.8)
**Amiodarone, n (%)**	28,325 (8.7)	8,624 (4.8)	33,564 (8.8)	12,233 (6.4)	28,467 (6.6)	31,286 (8.2)	66,004 (8.0)
**CCB, n (%)**	158,915 (48.7)	84,181 (47.1)	188,175 (49.2)	91,036 (47.3)	219,631 (51.0)	208,493 (54.6)	438,231 (52.9)
**Warfarin, n (%)**	47,636 (14.6)	19,418 (10.9)	83,765 (21.9)	21,163 (11.0)	85,644 (19.9)	70,204 (18.4)	146,442 (17.7)
**Acetylsalicylic acid, n (%)**	168,433 (82.3)	143,164 (80.1)	300,299 (78.5)	164,252 (85.3)	343,002 (79.6)	299,039 (78.3)	649,776 (78.4)
**NOAC, n (%)**	61,458 (18.8)	30,510 (17.1)	75,397 (19.7)	28,085 (14.6)	84,670 (19.6)	97,631 (25.6)	552,626 (20.3)
**OAD, n (%)**	149,037 (45.7)	66,736 (37.3)	177,054 (46.3)	83,080 (43.1)	197,303 (45.8)	167,593 (43.9)	389,477 (47.0)

ACE-i: angiotensinogen converting enzyme inhibitor; ARB: angiotensin-II receptor blockers; ARNI: angiotensin receptor blockers/neprilysin inhibitors; CCB: calcium channel blockers; NOACs: direct oral anticoagulants; MRA: mineralocorticoid receptor antagonists; OAD: oral antidiabetics; RAS: renin-angiotensin system; SGLT-2i: Sodium-glucose cotransporter two inhibitors.

**Table 3:** Advanced heart failure treatment methods used in Türkiye.

	Male (n = 1,314,224)	Female (n = 1,407,927)	Total (n = 2,722,151)
**Renal replacement therapy, n (%)**	77,284 (5.9)	63,764 (4.5)	141,048 (5.2)
**Cardiac and pulmonary rehabilitation, n (%)**	38,505 (2.9)	31,275 (2.2)	69,780 (2.6)
**Coronary artery bypass surgery, n (%)**	32,741 (2.5)	12,961 (0.9)	45,702 (1.7)
**ICD, n (%)**	18,143 (1.4)	4990 (0.4)	23,133 (0.8)
**Mitral valve replacement, n (%)**	3919 (0.3)	6172 (0.4)	10,091 (0.4)
**CRT, n (%)**	5852 (0.4)	3042 (0.2)	8894 (0.3)
**Aortic valve replacement, n (%)**	3513 (0.3)	2456 (0.2)	5969 (0.2)
**IABP, n (%)**	2974 (0.1)	1414 (0.1)	4388 (0.2)
**TAVI, n (%)**	1761 (0.1)	2368 (0.2)	4129 (0.2)
**ECMO, n (%)**	585 (0.0)	400 (0.0)	985 (0.0)
**Ventricular assisted device, n (%)**	344 (0.0)	57 (0.0)	401 (0.0)
**Percutaneous valve repair, n (%)**	215 (0.0)	133 (0.0)	348 (0.0)
**Heart transplantation, n (%)**	110 (0.0)	38 (0.0)	148 (0.0)
**Left atrial appendix closure, n (%)**	67 (0.0)	63 (0.0)	130 (0.0)

ECMO: Extracorporeal membrane oxygenation; IABP: Intraaortıc balloon pump; ICD: Implantable cardioverter-defibrillator; CRT: Cardiac resynchronization therapy; TAVI: transcatheter aortic valve replacement.
